# Multidimensional mechanisms of quercetin in diabetic kidney disease

**DOI:** 10.3389/fcell.2025.1705601

**Published:** 2026-02-25

**Authors:** Zhaoyuan Gong, Qianzi Che, Chuxuan Wang, Bin Liu, Mingzhi Hu, Haili Zhang, Ning Liang, Huizhen Li, Guozhen Zhao, Lijiao Yan, Tian Song, Lin Chen, Nannan Shi, Jing Guo

**Affiliations:** 1 Institute of Basic Research in Clinical Medicine, China Academy of Chinese Medical Sciences, Beijing, China; 2 Beijing University of Chinese Medicine, Beijing, China

**Keywords:** cellular senescence, diabetic kidney disease, literature review, network pharmacology, quercetin

## Abstract

Quercetin is a flavonoid compound that has demonstrated substantial potential in the treatment of diabetic kidney disease (DKD). However, there is still a lack of systematic research on the exact mechanism of action of quercetin. This review discusses the druggability, molecular targets, and signaling pathways of quercetin in DKD treatment. We retrieved the latest research on the pharmacological effects and mechanisms related to quercetin from PubMed and Scopus as of June 2025 (2012–2025). Evidence suggests that quercetin has the potential to eliminate senescent cells in DKD. Network pharmacology was used to predict the targets and pathways of quercetin in targeting cellular senescence to treat DKD. Using on existing research, it was further confirmed that quercetin can effectively act on hub target and pathway. The mechanism of quercetin therapy in DKD was summarized from three dimensions: inflammation, oxidative stress, and cell death. This review highlights the potential of quercetin for treating DKD by providing a biological basis for its mechanism of action and its use as a senolytic drug for this disease.

## 1 Introduction

Diabetic kidney disease (DKD) is one of the most common and serious complications of type 1 and type 2 diabetes, affecting approximately 40% of diabetic patients and imposing a huge burden on public health (Bell et al., 2015; Alicic et al., 2017; Chesnaye et al., 2024). Over the past 2 decades, the incidence of DKD has increased steadily worldwide (Harding et al., 2019; Naik et al., 2022). The clinical features of DKD include glomerular filtration rate reduction, glomerulosclerosis, tubulointerstitial fibrosis, and atrophy (Remuzzi and Bertani, 1998; Rao Kondapally Seshasai et al., 2011). DKD can lead to progressive renal failure, primarily resulting in thylakoid expansion and thickening of the glomerular basement membrane (Hu et al., 2022). In addition to its negative effects on patient health, DKD exerts a heavy burden on society.

The pathogenesis of DKD is extremely complex. The disease is driven by a series of disordered metabolic, hemodynamic, inflammatory, and fibrotic processes (Tuttle et al., 2022). The core mechanisms mainly include: 1) chronic hyperglycemia, leading to metabolic disorders (Doumani et al., 2025); 2) oxidative stress (Jin et al., 2023); 3) a chronic inflammatory response (Rayego-Mateos et al., 2020); 4) cellular senescence (Chen L. et al., 2021); and 5) various modes of cell death models (Yang C. et al., 2023). Among these processes, the role of senescence in DKD pathogenesis has attracted considerable attention. The onset of DKD occurs as a result of concurrent continuous injury to the kidney from diabetes and cellular senescence-induced impairment of tissue repair (Wei et al., 2024). In addition, the available treatment methods for DKD are limited. Current clinical management programs are insufficient to reduce the morbidity and poor prognosis associated with DKD; thus, new treatment options and concepts must be developed (Li et al., 2025). Determining the pathogenesis and therapeutic options for DKD is critical for preventing progressive renal function loss (Chen L. et al., 2021). One potentially effective approach is seeking potential drugs targeting cellular senescence.

**FIGURE 1:**
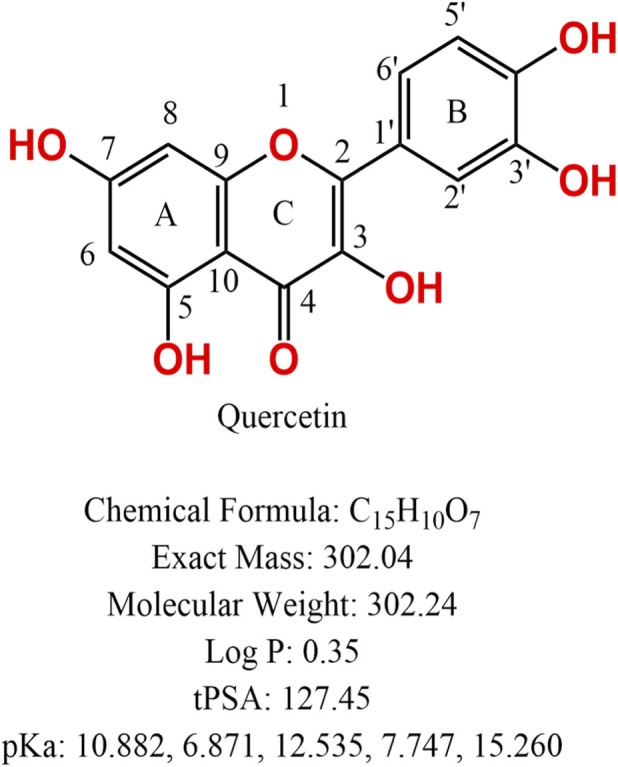
Structure and chemical properties of quercetin.

In recent years, the therapeutic effects and mechanisms of traditional Chinese medicine prescriptions and their active components in DKD have received widespread attention in new drug development (Liu X. J. et al., 2022). Quercetin (also known as 3,3′,4′5,7-pentahydroxyflavone) is a natural flavonoid compound (Figure 1) that exerts various biological functions, including antidiabetic (Yan et al., 2023), anti-inflammatory (Li et al., 2016), antioxidant (Martín et al., 1998), and anticancer effects (Reyes-Farias and Carrasco-Pozo, 2019). Evidence suggests that quercetin has the potential to eliminate senescent cells in DKD (Hickson et al., 2019). Furthermore, quercetin has shown efficacy in the management of diabetes; however, its exact mechanism remains unclear (Anand David et al., 2016).

Therefore, in this review, we used network pharmacology and molecular docking to identify potential therapeutic targets, biological functions, and signaling pathways of quercetin in treating DKD by targeting cellular senescence. Subsequently, we used keywords such as “cellular senescence”, “diabetic nephropathy”, “hub genes”, and “key pathways of network pharmacology” in the PubMed and Scopus databases to review the literature, focusing on the research published in the past decade. Using emerging research evidence, we explored how quercetin plays an important role in combating cellular senescence in the treatment of DKD, providing new insights for future research on quercetin and DKD treatment. This study provides scientific evidence for quercetin as a therapeutic intervention for DKD.

## 2 Physicochemical properties and druggability of quercetin

### 2.1 Physicochemical properties

Quercetin, a brilliant citron yellow needle crystal with lipophilic (fat-soluble) properties, is relatively soluble in alcohol and lipids, completely insoluble in cold water, and poorly soluble in hot water (El-Saber Batiha et al., 2020). Dimethyl sulfoxide or N, N-dimethylformamide are used to solubilize quercetin in most studies (Demir et al., 2020); some researchers have used corn oil (Yurteri et al., 2023). Quercetin exists in a glycoside form (i.e., bound to one, two, or more saccharide molecules) in plants, vegetables, and fruits, which increases its water solubility (Ross and Kasum, 2002). The glycosylation of quercetin usually occurs as a substitute for the hydroxyl radical at position 3; however, it may also occur at positions 3′, 4′, or 7′ (Kapešová et al., 2019).

### 2.2 Bioavailability, absorption, metabolism, and safety

The relatively low bioavailability of quercetin limits its clinical application and therapeutic efficacy. Its gastrointestinal tract absorption is also considered very low, with an oral bioavailability of 1% in humans. Research has demonstrated that the daily intake of quercetin among Greek adults is approximately 20 mg, and the total plasma concentration of both free and conjugated quercetin ingested in the short term ranges from 72 to 193 nmol/L in humans (Manzoor et al., 2021). The highest level of quercetin in plasma is reached after 0.7–7.0 h (Wang et al., 2016).

Following oral administration, quercetin is not absorbed in the mouth or stomach but in the small intestine and liver, where it is further metabolized (Zhu et al., 2024a). In the small intestine, quercetin undergoes phase II metabolism after absorption, including glucuronidation, methylation and sulfation (Zhang et al., 2007; Luca et al., 2020). In the liver, quercetin and its metabolites undergo phase I/II metabolism. Cytochrome P450 is responsible for phase I metabolism (e.g., oxidation, reduction, hydrolysis, and hydration) (Hodek et al., 2002), and phase II metabolism involves various conjugation reactions, including glucuronidation, sulfation, and methylation (Zhu et al., 2024a). Finally, quercetin and its metabolites are eliminated through the kidneys, feces, and respiratory system after exerting their effects (Di Petrillo et al., 2022). Research has confirmed that quercetin has a half-life of 11–28 h. Therefore, the low bioavailability of quercetin results from poor water solubility due to increased lipophilicity, reduced gastrointestinal absorption, instability in the stomach and intestine, a short half-life, low permeability, oxidative degradation, and the high efficiency of the first hepatic passage (Zhu et al., 2024a). In the past decade, numerous delivery systems have been developed to enhance the bioavailability of quercetin, including lipid-based carriers (Ozkan et al., 2020), polymer nanoparticles (Xu et al., 2019), inclusion complexes (Nagaraj et al., 2024), micelles (Sun et al., 2023), and conjugated capsules (Wang and Morris, 2005). In addition, new quercetin derivatives have been used to address the limitations of quercetin, including quercetin hybrids, C-substituted derivatives, and imine, selenone, and thienone derivatives (Alizadeh and Ebrahimzadeh, 2022). However, significant uncertainty arises with both delivery strategies and derivative designs when translated into clinical applications (Zhao et al., 2025).

Table 1 summarizes the adverse reactions reported across all clinical studies evaluating quercetin safety. Quercetin is generally well-tolerated in human clinical studies. In 2010, quercetin supplements were added to the Food and Drug Administration’s Generally Recognized as Safe (GRAS) list as a supplementary ingredient in foods and beverages up to 500 mg per serving (Dabeek and Marra, 2019). Most *in vivo* animal studies have shown that quercetin is a safe compound without any carcinogenic effects (Rietjens et al., 2005; Harwood et al., 2007). Overall, long-term safety data for high-dose quercetin are lacking in the quercetin safety literature (Harwood et al., 2007).

**TABLE 1:** Adverse reactions reported in clinical studies.

Subjects			Quercetin form	Dosage (mg)	Duration (weeks)	Mean fasting plasma concentrations	References
Total (Men/Women)	Age (Mean ± SD)	Disease status					
93 (42/51)	45.1 ± 10.53	Overweight, obese, and stage I hypertension	Quercetin	150	6	Increase from 71 to 269 nmol/L	Egert et al. (2009)
49 (49/0)	59.4 ± 0.9	APOE3/3 (n = 19) and APOE4 (n = 30)	Quercetin	150	8	Increase from 121.9 ± 7.5 to 193.8 ± 20.4 nmol/L	Pfeuffer et al. (2013)
20 (Quercetin group)	26.1 ± 1.8	Cyclists	Quercetin	1,000	6	304 ± 71 μg/L after 3 weeks	McAnulty et al. (2008)
30 (20/10)	25–65	Chronic hepatitis C virus infection	Quercetin	250–5,000	4	2,256 ± 1595 μg/L after 4 weeks	Lu et al. (2016)

## 3 Antisenescence mechanism of quercetin in the treatment of diabetic kidney disease

Recently, the role of cellular senescence in DKD has attracted widespread attention. Studies have confirmed that in the context of diabetes, various pathogenic stimuli, including hyperglycemia and the accumulation of advanced glycation end products (AGEs), can induce renal parenchymal cells to undergo cellular senescence (Liu et al., 2015; Jia et al., 2020). Cellular senescence may also regulate microangiopathy in diabetes and exacerbate oxidative stress in microvascular endothelial cells under high glucose conditions (Abouhish et al., 2020) Furthermore, senescence can establish a pathogenic positive feedback loop, promote the development and accumulation of senescent cells in the organism, and accelerate the pathophysiological process of diabetes (Palmer et al., 2015; Tsai et al., 2018). Senescent cells are characterized by permanent cell cycle arrest and loss of proliferative capacity (Gorgoulis et al., 2019). The four most widely recognized types of cellular senescence are replicating senescence (Harley et al., 1990), stress-induced premature senescence (Prieur and Peeper, 2008), oncogenic-induced senescence (Serrano et al., 1997), and developmentally programmed senescence (Muñoz-Espín et al., 2013). The triggers of cellular senescence include but are not limited to telomeric erosion, induction of the DNA damage response, epigenetic changes, genomic instability, mitochondrial dysfunction, reactive metabolites, oxidative stress, inactivation of certain tumor suppressor genes, oncogenic and therapy-induced stress, and viral infections (Narita et al., 2003; Campisi, 2005; Di Micco et al., 2006; Passos et al., 2010; Wiley et al., 2016; Robinson et al., 2018; Camell et al., 2021; Wiley and Campisi, 2021). Detecting the molecular and morphological characteristics of senescent cells can distinguish them from normal cells (Zhang et al., 2022). Morphological changes that serve as signs of aging include 1) the expansion and irregular shape of the cell body; 2) changes in the composition of the plasma membrane; 3) increased lysosome content; 4) mitochondrial accumulation; and 5) absence of the nuclear layer structural protein LaminB1 (Hernandez-Segura et al., 2018). The cellular senescence program is initiated by the p53/CDKN1A (p21cP, hereafter p21) and CDKN2A (p16INK4a, hereafter p16)/Rb tumor suppressor pathways (Herranz and Gil, 2018). Senescent cells retain metabolic activity and can acquire senescence associated secretory phenotypes (SASPs) that promote inflammation, tissue destruction, and apoptosis (Coppé et al., 2008). SASPs include the secretion of various molecules, including cytokines, chemokines, and growth factors, and are considered a possible source of inflammatory factors in DKD (Prattichizzo et al., 2018). The pathogenic role of senescence in DKD and the treatment targeting this process have received increasing attention (López-Otín et al., 2023).

### 3.1 Cellular senescence: eliminating senescent cells

The role of cellular senescence in DKD has attracted widespread attention, and involves multiple mechanisms including telomere shortening, DNA damage, epigenetic modifications, and mitophagy deficit (Xiong and Zhou, 2019; Shen et al., 2022). The increased accumulation of senescent cells in the kidneys of diabetic patients impairs kidney repair capacity and leads to the secretion of proinflammatory and profibrotic cytokines and growth factors, resulting in inflammation and fibrosis (Wei et al., 2024). Quercetin plus dasatinib has been found to effectively eliminate senescent cells in DKD patients (Zhu et al., 2015). In an open-label Phase I pilot study, researchers administered a combination of dasatinib and quercetin to nine DKD patients. The patients’ adipose tissue senescent cell burden was reduced within 11 days, with decreases in cells expressing p16^INK4A^ and p21^CIP1^, cells with senescence-associated β-galactosidase activity, and adipocyte progenitors with limited replicative potential. Furthermore, there was a decrease in adipose tissue macrophages and crown-like structures that were attracted, anchored, and activated by senescent cells in the patients’ bodies. Circulating pro-apoptotic SASPs, including IL-1α, IL-6, MMPs-9, and IL-12, were also reduced by quercetin plus dasatinib (Hickson et al., 2019). In aged mice, treatment with dasatinib plus quercetin significantly reduced the senescent cell (p16 and p21 expression) and inflammatory (Cxcl1, Il1β, Il6, Mcp1 and TNF-α expression) burden in the small and large intestine (Saccon et al., 2021). Few studies have investigated the senolytic effects of quercetin alone. Recently, Jimenez et al. demonstrated that quercetin could improve the senescence phenotype of rat cardiomyocytes, which was associated with increased mitochondrial-endoplasmic reticulum contact sites, reduced distance between the two organelles, and decreased ROS production (Jiménez et al., 2025). Quercetin has also been found to significantly increase the gene and protein expression of Senescence marker protein 30 (SMP30), thereby reducing the complications of diabetes in kidneys and preventing kidney cell aging (Abharzanjani and Hemmati, 2021). Overall, further research is needed into the effects of quercetin on DKD through anti-cellular senescence mechanisms, and its individual therapeutic effect remains to be elaborated.

### 3.2 Potential mechanisms of the antisenescence effect of quercetin in diabetic kidney disease

As bioinformatics rapidly develops, network pharmacology, which integrates large databases with systems biology and pharmacology, has become a powerful tool for describing drug treatment mechanisms from the molecular to the pathway level (Guo et al., 2019; Zhou et al., 2020). Network pharmacology can reveal the mechanisms of drug action in disease by depicting complex “drug–gene–target–disease” interactive networks. This method has become a promising approach to accelerate drug development (Huang et al., 2019). In addition, network topology analysis enables the exploration of key nodes and pathways.

#### 3.2.1 Potential cellular senescence targets and network identification of quercetin in the treatment of diabetic kidney disease

**FIGURE 2:**
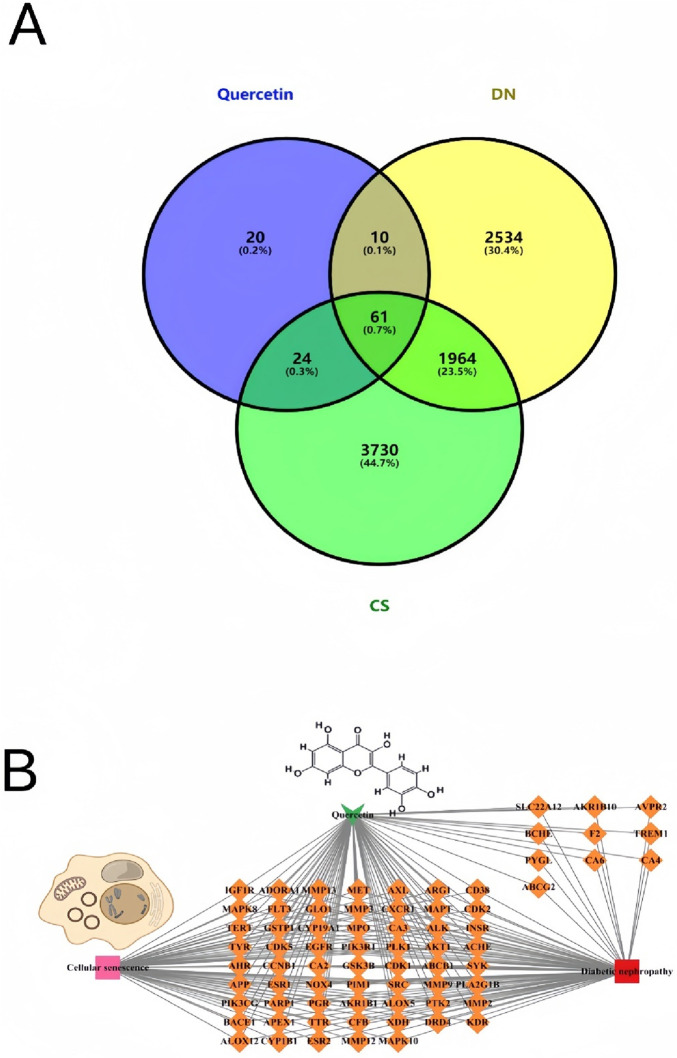
Quercetin targets and networks for treating DKD through cellular senescence. **(A)** Venn diagrams showing targets numbers of Quercetin, DKD, and cellular senescence (CS). **(B)** Quercetin–cellular senescence–diabetic nephropathy -targets network. Green arrows represent the Quercetin, red circles node are diabetic nephropathy, pink circles node are cellular senescence, orange circles node are the predicted targets. The edges represent the interaction between compounds and targets.

We used the PubChem database (https://pubchem.ncbi.nlm.nih.gov/) to obtain the SDF and SMILES structures of quercetin. Subsequently, we retrieved its targets using Swiss Target Prediction (http://www.swisstargetprediction.ch/) with SMILES and probability*(P) ≥ 0.1 as a screening indicator (Daina et al., 2019). Furthermore, we predicted potential targets by inputting SDF structures into the PharmMapper database (http://www.lilab-ecust.cn/pharmmapper/) using Norm Fit value 8 as a screening indicator (Liu et al., 2010). Finally, we used the Universal Protein database (Uniprot, http://uniprot.org/) to verify the targets (Apweiler et al., 2004). A total of 115 related targets of quercetin were obtained from the PharmMapper and SwissTargetprediction databases (Supplementary Table S1). We collected DKD-related targets by searching Genecards (https://www.genecards.org/) and Online Mendelian Inheritance in Man (OMIM, https://omim.org/#) using “diabetic nephropathy” as the keyword (Amberger et al., 2015; Safran et al., 2022). Then, we merged the results and removed duplicate targets. The cellular senescence-related targets were obtained using the Genecards and Gene Set Enrichment Analysis (GSEA; https://www.gsea-msigdb.org/) databases (Subramanian et al., 2005). We searched two databases for DKD-related research reports to identify potential targets. A total of 4,637 targets in DKD were screened, of which 4,376 were from the Genecards database, and 261 were from the OMIM database. After expurgating duplicate targets, 4,573 targets related to DKD were screened out (Supplementary Table S2). We searched two databases for research reports on cellular senescence to identify potential targets. A total of 5,991 targets of cellular senescence were screened, of which 5,779 were from the Genecards database and 212 from the GSEA database (Human Gene Set: REACTOME_CELLULAR_SENESCENCE). After expurgating duplicate targets, 5,779 targets related to cellular senescence were screened out (Supplementary Table S3). A Venn diagram was generated to identify potential quercetin targets of cellular senescence in DKD treatment. Excel software was used to screen the intersected relevant targets for quercetin, DKD-related targets, and cellular senescence-related targets. Then, the “quercetin–target–cellular senescence–disease” network was constructed using Cytoscape 3.9.1. This network took diseases, drugs, components, and related targets as nodes, and their interrelationships as edges. Finally, 61 common targets were regarded as potential targets of quercetin against cellular senescence in DKD treatment (Figure 2A) (Supplementary Table S4). To elucidate the relationship between quercetin, targets, cellular senescence, and DKD, we further constructed a drug–target–cellular senescence–disease network using Cytoscape 3.9.1 software, comprising 74 nodes and 203 edges (Figure 2B).

#### 3.2.2 PPI network construction and identification of hub targets

**FIGURE 3:**
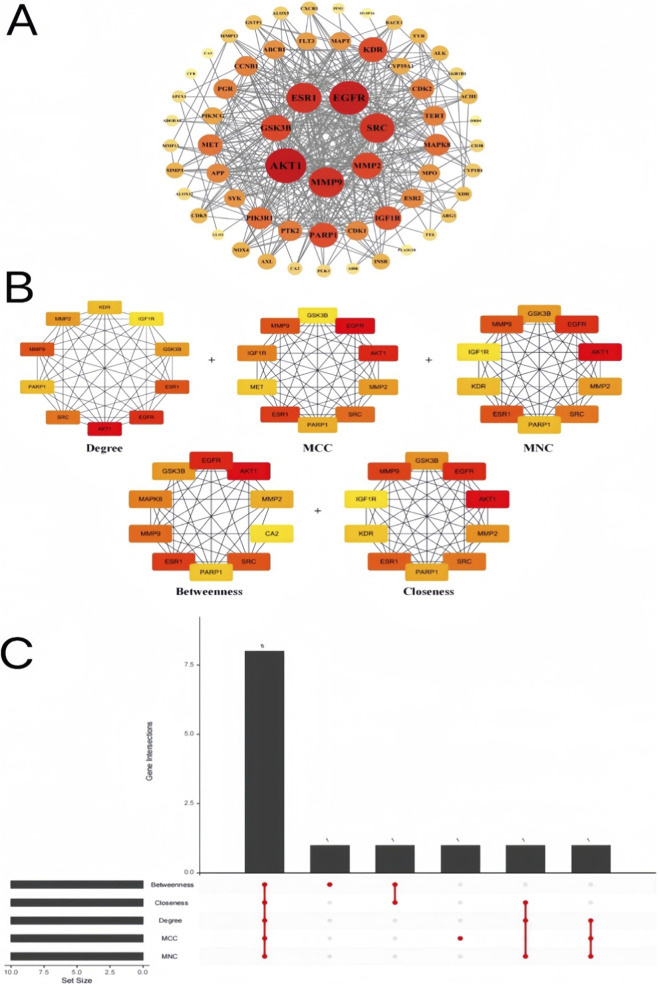
PPI network construction and identification of hub targets. **(A)** The PPI network construction. The size of nodes is directly proportional to the degree of targets in the network. The color was from pale yellow to red, and the corresponding degree gradually larger. **(B)** Using CytoHubba plugin to select hub genes from PPI network. The color of the nodes was from pale yellow to red, and the corresponding degree gradually became larger. **(C)** Upset diagram showing five calculation methods for hub genes. The horizontal bars show the size of the gene set for each algorithm, and the vertical bars show the size of the intersections. The eight hub targets (e.g., AKT1, EGFR) were consistently identified by multiple algorithms, indicating high confidence in their critical roles.

We used the String database (https://string-db.org) to construct the PPI network of the intersection targets by setting the species as *Homo sapiens* and the minimum required interaction score as 0.40 (Szklarczyk et al., 2021). The hub genes were screened by the five algorithms in the CytoHubba plugin. The UpSet package in R was used to identify the intersection of hub genes. We uploaded 61 targets into a String database to construct a PPI network with 61 nodes and 392 edges, yielding an average node degree of 12.9 (Figure 3A). Node size and color intensity are proportional to the degree of connection (the number of interactions a protein has). The network analysis revealed that EGFR, AKT1, MMP9, ESR1, MMP2, and SRC were the core network proteins. To identify the hub targets, we used CytoHubba to set the calculation methods of betweenness (measures how often a node acts as a bridge along the shortest path between two other nodes; high-betweenness nodes are critical for information flow), closeness (measures how close a node is to all other nodes in the network; high-closeness nodes can quickly influence the entire network), degree (the simplest measure, indicating the number of direct connections a node has; high-degree nodes are highly interconnected), maximum neighborhood component (MNC; identifies nodes within large, densely connected regions), and maximal clique centrality (MCC; identifies nodes that are part of the most densely interconnected sub-networks) (Figure 3B). Ranking was performed using five algorithms that capture different aspects of a node’s importance in a network. We conducted an UpSet diagram analysis to obtain the intersection of hub targets for multiple calculation methods (Figure 3C). We ultimately identified eight hub targets, including AKT1 (RAC-alpha serine/threonine-protein kinase), EGFR (Epidermal growth factor receptor), ESR1 (Estrogen receptor), SRC (Proto-oncogene tyrosine-protein kinase Src), MMP9 (Matrix metalloproteinase-9), GSK3β (Glycogen synthase kinase-3 beta), MMP2 (72 kDa type IV collagenase), and PARP1 (Poly [ADP-ribose] polymerase 1).

#### 3.2.3 Gene ontology and kyoto encyclopedia of genes and genomes analyses

**FIGURE 4:**
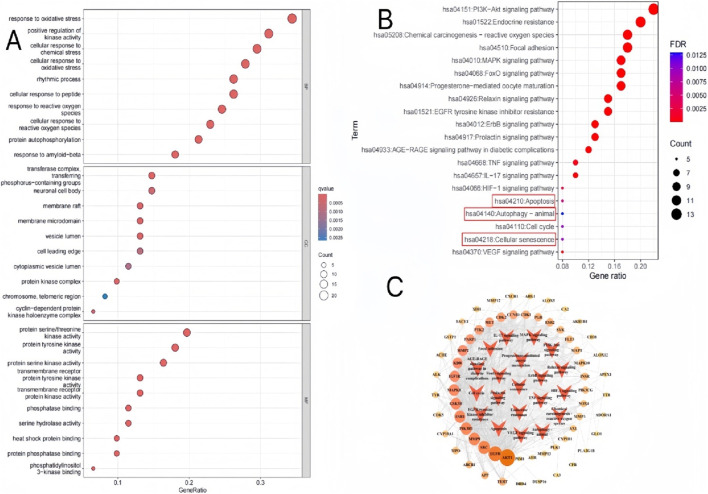
GO and KEGG enrichment analysis. **(A)** The classification circle plot showed the GO result. **(B)** The circle plot showed the KEGG result. **(C)** Target-KEGG pathway network. The color of the nodes was from pale yellow to red, and the corresponding degree gradually became larger. The red arrow represented the pathway.

Gene Ontology (GO) and Kyoto Encyclopedia of Genes and Genomes (KEGG) enrichment analyses were performed using gmt files downloaded from the GSEA platform (http://www.gsea-msigdb.org/). Cytoscape 3.9.1 was used to construct the quercetin–target pathway network. The GO enrichment and KEGG pathway analysis results were visualized in R. GO and KEGG enrichment analyses were performed to identify key biological processes and representative signaling pathways of quercetin in combating cellular senescence to treat DKD. GO analysis revealed that the 61 genes were enriched in 1670 GO entries: 1,517 biological processes (BP), 47 cellular components (CC), and 105 molecular functions (MF) (*p* < 0.05) (Supplementary Table S6). The top 10 BP, CC, and MF pathways are shown in Figure 4A. In the BP analysis, the relevant targets were mainly enriched in response to oxidative stress, cellular response to oxidative stress, and response to reactive oxygen species. In the CC analysis, the relevant targets were mainly centered on transferase complex, transferring, phosphorus-containing groups, neuronal cell body, and membrane raft. In the MF analysis, the potential targets were primarily focused on protein serine/threonine kinase activity, protein tyrosine kinase activity, and phosphatase binding.

The KEGG enrichment analysis identified 127 significantly enriched signaling pathways (*p* < 0.05) (Supplementary Table S5). The top 20 significantly enriched signaling pathways are shown in (Figure 4B), including the PI3K-AKT signaling pathway, chemical carcinogenesis-reactive oxygen species, MAPK signaling pathway, apoptosis, autophagy—animal, and cellular senescence. Subsequently, we visualized the leading 20 signaling pathways and their respective targets, as shown in Figure 4C.

#### 3.2.4 Molecular docking verification of the interaction between quercetin and hub targets

**FIGURE 5:**
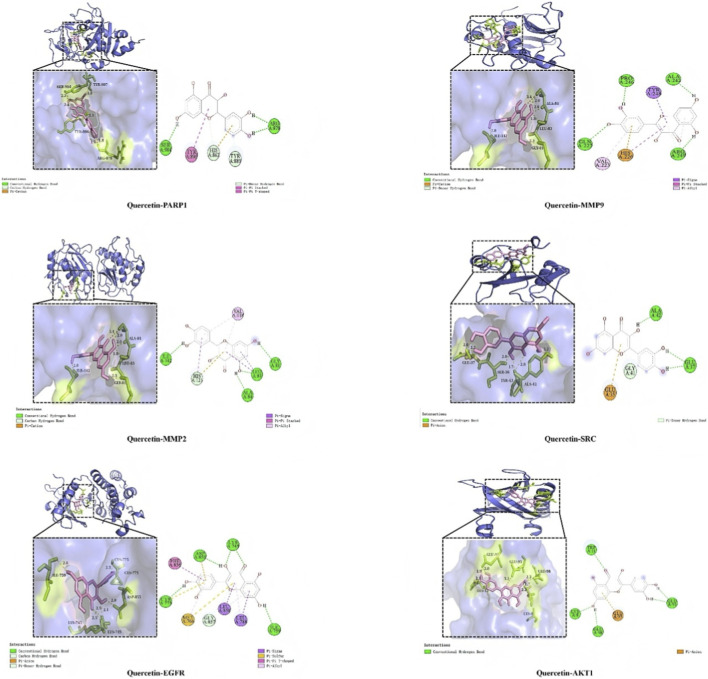
Molecular docking analysis between quercetin and hub targets. The 3D molecular docking structures of key compounds and eight key target genes.

We obtained the 3D structures of quercetin and the hub genes from the PubChem and Protein Data Bank (PDB) (http://www.rcsb.org/) databases (Berman et al., 2000). Molecular docking was performed using AutoDock Vina to validate the interactions of quercetin with the target genes, and the top four docking poses were selected and visualized in PyMOL. We validated quercetin’s binding affinity to hub targets using molecular docking. The top complexes with strong quercetin binding ability were MMP9 (−9.72 kcal/mol), EGFR (−8.47 kcal/mol), MMP2 (−8.05 kcal/mol), PARP1 (−7.39 kcal/mol), ESR1 (−6.65 kcal/mol), AKT1 (−6.53 kcal/mol), GSK3β (−6.37 kcal/mol), and SRC (−6.09 kcal/mol) (Figure 5). Thus, quercetin exhibited strong binding to hub targets and could bind stably.

### 3.3 Mechanism of action between quercetin and signaling pathways of hub genes

**FIGURE 6:**
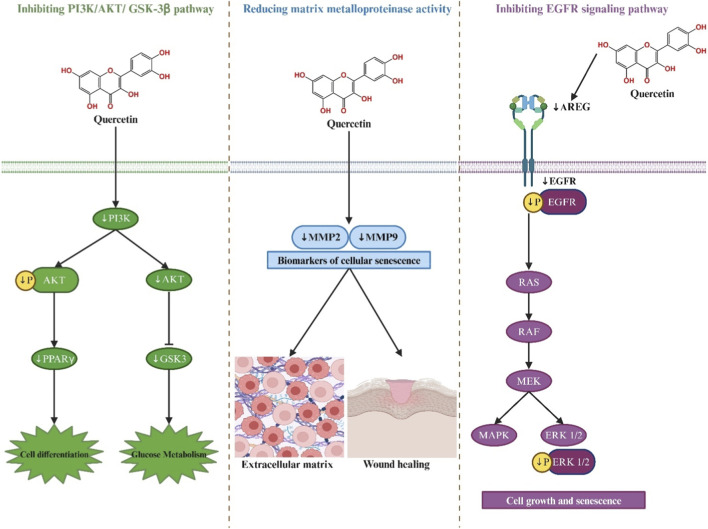
The mechanism of action between quercetin and signaling pathways of hub genes.

The network pharmacology results revealed that quercetin may combat cellular senescence and treat DKD through hub targets (AKT1, EGFR, ESR1, SRC, MMP9, GSK3β, MMP2, and PARP1). Thus, we further investigated the role of quercetin in hub target-related pathways. The mechanism of action between quercetin and hub gene signaling pathways is shown in Figure 6.

#### 3.3.1 Quercetin inhibits PI3K/AKT/GSK-3β pathway activation

The P13K–AKT pathway is important in insulin signal transduction. The response to insulin is reduced in diabetes, and impaired glucose stability is one of the key pathogenic mechanisms (Fang et al., 2022). Changes in the protein level and activity of key proteins in the PI3K–AKT pathway may damage the pathway and impair insulin signal transduction, leading diabetes occurrence and development (Chen et al., 2023). AKT1, also known as AKT kinase, is one of three closely related serine/threonine-protein kinases (AKT1, AKT2, and AKT3) and is expressed in insulin-sensitive tissues, such as the liver, skeletal muscles, and adipose tissue (Huang et al., 2018).

In diabetes, the overexpression or enhancement of AKT activity has been found to increase glucose uptake in skeletal muscle, thus maintaining euglycemia (Zhang et al., 2019). Meanwhile, the AKT gene family is the main regulatory factor for cell proliferation and senescence (Bayer et al., 2022). AKT can regulate many processes, including metabolism, proliferation, cell survival, growth, and angiogenesis (Cartlidge et al., 2005; Walia et al., 2019). Activating AKT can increase the survival rate of pancreatic beta cells (Alwhaibi et al., 2019). Li et al. treated 3T3-L1 preadipocytes with quercetin and found that it may inhibit PI3K–AKT pathway activation by suppressing PI3K and phosphorylated AKT (p-AKT) protein expression and inhibit preadipocyte differentiation by reducing PPARγ protein expression (Li et al., 2020). In another study, quercetin was found to significantly enhance p-AKT expression and significantly reduce acetylated AKT expression in the liver of STZ-induced diabetic rats (Peng et al., 2017).

GSK3β plays a central role in DKD pathogenesis (Liang et al., 2020). I Insulin resistance results from impaired signal transduction in the insulin–PI3K–AKT signaling pathway, leading to increased GSK-3β levels (Zhang et al., 2018). GSK3β has been identified as a potential novel therapeutic target in DKD mice as it is overactive in glomerular podocytes and is associated with IRS-1 hyperphosphorylation, impaired Nrf2 response, and premature senescence (Chen et al., 2024). The specific resection of GSK-3β in podocytes significantly alleviated podocyte senescence and glomerular aging in mice (Kreidberg and Schumacher, 2022). Abdou et al. found that quercetin could significantly reduce the level of GSK-3β in the kidney in a rat diabetes model (Abdou and Abd Elkader, 2022). Quercetin also reduces the expression of PI3K, FOXO1, AKT, AMPK and GSK-3β in the cerebral cortex of diabetic rats. In addition, treatment with quercetin reduced the production of proinflammatory cytokines TNF-α and NF-κB and downregulated the levels of IL-1β, iNOS, APP, and PPARγ (Newairy et al., 2024).

#### 3.3.2 Quercetin reduces matrix metalloproteinase activity

Matrix metalloproteinase (MMPs) are crucial in the leukocyte migration and local extracellular matrix (ECM) proteolysis (Wilhelm et al., 1989). Decreasing MMPs increases extracellular matrix growth and enhances wound healing in diabetes (Zhang et al., 2020). ROS production is necessary for the synthesis of MMPs, which disrupt ECM remodeling (Nelson and Melendez, 2004), and is a primary driver of cellular senescence (Guillaumet-Adkins et al., 2017; Höhn et al., 2017). This dual role positions ROS as a critical link between ECM dysregulation and the accelerated aging process observed in DKD.

MMP2 and MMP9 are biomarkers of senescence. In one study evaluating the association between plasma levels of 28 SASP proteins and mortality risk, MMP2 was identified as the senescence biomarker most closely associated with increased mortality risk (Lin et al., 2020; St. Sauver et al., 2023). Gelatinases (MMP2 and MMP9) are particularly involved in the wound healing process. MMP9 and MMP2 also play a key role in the treatment of diabetes and its complications. Overproduction of MMP9 will lead to excessive degradation of the ECM in diabetes patients and delayed wound healing (Ayuk et al., 2016). Quercetin has been found to significantly reduce the levels of MMP9 and VEGF mRNA and protein in the retina of diabetes rats (Chen et al., 2017). Bagheri et al. evaluated the renal protective effect of quercetin using a rat model of renal ischemia/reperfusion injury. The results showed that quercetin significantly reduced the expression levels of MMP2 and MMP9 (Bagheri et al., 2022). In addition, quercetin significantly inhibits TNF-α-induced upregulation of MMP9 and cell migration (Hwang et al., 2009).

#### 3.3.3 Quercetin inhibits the EGFR and MAPK signaling pathways

EGFR is widely expressed in glomeruli, proximal tubules, and collecting ducts (Zhang et al., 2014), and activated EGFR is a key mechanism for podocyte dysfunction and loss during DKD development (Chen et al., 2015). These intracellular pathways include the SRC kinase and PI3K pathways. The EGFR signaling pathway is known to be associated with cell growth and senescence (Groveman et al., 2023). Epidermal growth factor (EGF) deficiency disrupts cell proliferation and causes cells to enter a senescent state (Alexander et al., 2015). EGFR activation has been found to play an important role in activating the pathway mediating podocyte injury and loss in diabetic nephropathy (Skibba et al., 2016). Liu et al. confirmed the inhibitory effect of quercetin on the EGFR pathway in diabetic mice. The results showed that quercetin could downregulate the expression of phosphorylated EGFR and phosphorylated ERK in these animals (Liu Y. et al., 2022).

Quercetin acts on membrane EGFR and TNFR1 by promoting the release of VEGFA and TNF, thereby affecting the activation of Ras, Raf, and MEK and the phosphorylation of MAPK1 and MAPK3, leading to changes in angiogenesis, cell apoptosis, and cell proliferation (Zu et al., 2021). Amphiregulin (AREG) is a low affinity EGFR ligand. In one study, quercetin was shown to inhibit ERK1/2 and AKT phosphorylation in human proximal tubular epithelial cells (HK-2) stimulated by transforming growth factor beta 1 (TGF-β1). In *in vivo* experiments, quercetin treatment partially eliminated AREG upregulation and effectively inhibited EGFR (Wang et al., 2023).

MAPK is a well-recognized canonical proinflammatory signaling pathway. In DKD, MAPK family proteins are activated, and the phosphorylation levels of Erk1/2, JNK, and P38 increase significantly (Ma L. et al., 2021). P38 MAPK is believed to play a role in insulin-stimulated glucose uptake (Somwar et al., 2002). Dhanya et al. found that after quercetin pretreatment, the expression of MAPK was upregulated by more than five times, and that of P38MAPK doubled; P38MAPK phosphorylation also increased (Dhanya et al., 2017). Furthermore, it has been shown that quercetin inhibits H_2_O_2_-induced phosphorylation of p38 MAPK in INS-1 insulin-secreting β-cells. The protective effect of quercetin is due to ERK1/2 hyperactivation, which may be caused by the opening of L-type calcium channels (Youl et al., 2014). However, similar studies are limited. Further research is needed to reveal the inhibitory effect of quercetin on MAPK in diabetes models.

#### 3.3.4 The effects of quercetin on PARP-1, SRC, and ESR1 signaling require additional research

PARP1 is a nuclear protein involved in DNA repair. Increased DNA damage resulting from hyperglycemia in diabetes leads to an increase in PARP1 expression (Szabó et al., 2002; Pacher and Szabó, 2005). PARP1 promotes vascular smooth muscle cell proliferation and migration, accelerating the process of hyperglycemia-induced neointimal hyperplasia. Thus, PARP1 activation plays a key role in the pathogenesis of diabetes complications (Wang et al., 2021). Under physiological conditions, PARP1 protects cells from senescence; under pathological conditions, it induces senescent cell apoptosis to prevent stress (Mukhopadhyay et al., 2017). PARP loss is one of the metabolic drivers of senescence, leading to the loss of oxidized nicotinamide adenine dinucleotide (NAD^+^) and triggering senescence (Wiley and Campisi, 2021). Zhao et al. found that targeted PARP1 activation could alleviate diabetes-induced cellular senescence and promote wound healing in diabetes (Zhao et al., 2022). Additionally, quercetin could inhibit PARP1 levels, increase SIRT3 levels, protect mitochondrial function by regulating the SIRT3/PARP-1 pathway, treat spontaneously hypertensive rats, and inhibit cardiac hypertrophy (Chen W. J. et al., 2021). Furthermore, in an acute kidney injury model using HK-2 cells, quercetin was shown to decrease PARP1 cleavage while increasing the phosphorylation of AKT (Ser473), pSTAT3 (Tyr705), and FoxO3a (Thr32) (Andreucci et al., 2018). Chamgordani et al. constructed a diabetic rat model and found that the expression level of PARP1 mRNA was significantly increased in diabetic rats, and quercetin reversed this effect (Chamgordani et al., 2023).

SRC proteins are SRC family kinases that play important roles in cellular metabolism, growth, development, and differentiation. The SRC pathway triggers the activation of numerous genes, including those related to glucose metabolism and angiogenesis (Byeon et al., 2012). As mentioned earlier, SRC is involved in the PI3K–AKT signaling pathway and EGFR pathway. SRC directly regulates PI3K–AKT signaling pathway activity, thereby improving glucose metabolism dysfunction (Su et al., 2009). SRC also plays a key role in lipid metabolism and atherosclerosis, which are crucial to the cardiovascular health of type 2 diabetes patients (Ye et al., 2025). SRC is an upstream regulator of the balance between senescence and apoptosis. Anerillas et al. found that SRC inhibitors prevent the accumulation of senescent cells in mouse kidneys and increase apoptosis after treatment with doxorubicin (Anerillas et al., 2022). SRC inhibitors could alleviate the accumulation and harmful effects of senescence cells, which may improve the outcomes of treatments such as renal transplantation (Wei et al., 2017). Quercetin reduces the level of phosphorylated SRC protein and inhibits SRC/Stat3 signaling in human squamous carcinoma cell line A431-P (Fan et al., 2019). Furthermore, quercetin reduces phosphorylated SRC/SRC and alleviates cell damage in rat intestinal epithelial cells (Fan et al., 2021). However, the role of SRC in cellular senescence and the effect of quercetin on SRC in DKD remains unclear.

Finally, research on the interaction between quercetin and ESR1 and the role of ESR1 in cellular senescence is extremely limited. Estrogen receptor (ER) signaling pathways may contribute to various kidney diseases, including DKD (Ma H. Y. et al., 2021). Overexpression of ER-α36 partially restores PI3K–AKT signaling, attenuates cellular injury, and reverses high-glucose-induced epigenetic changes at the PTEN locus (Yu et al., 2025). Therefore, based on the known biology of these targets in senescence and DKD, we predict that quercetin treatment may inhibit PARP1 activity in renal tissue, reduce NAD^+^ consumption, help maintain cellular energy homeostasis, improve mitochondrial function, alleviate oxidative stress, inhibit cellular senescence, and treat DKD. Furthermore, we predict that quercetin treatment may promote the clearance of senescent cells by inhibiting SRC, thereby delaying the progression of DKD. Finally, given the current limited research on the role of ESR1 in the senescence of DKD cells, the prediction of its effect is more exploratory. We predict that quercetin treatment may inhibit the initiation of the cellular senescence program and treat DKD by up-regulating or stabilizing the expression of ESR1. Future studies are needed on the role of quercetin–hub gene interactions on cellular senescence in DKD treatment.

## 4 Other mechanisms of quercetin in the treatment of diabetic kidney disease

Quercetin could regulate DKD through various pathways, including apoptosis and autophagy. The specific mechanisms of quercetin in treating DKD are as follows.

### 4.1 Inhibiting apoptosis

Apoptosis is an important factor in the occurrence of DKD, and various factors such as glycolipid toxicity, angiotensin, and glycosylation end products, induce podocyte apoptosis through different pro-apoptotic pathways, leading to the occurrence of DKD (Bhatt et al., 2016). Quercetin exerts anti-cellular damage, anti-apoptosis, and protective effects against streptozotocin-induced nephrotoxicity in rat kidney epithelial cells (DH et al., 2024). Liu et al. established DKD models using Lepdb/Lepdb (db/db) mice and high-glucose (HG)-induced mouse podocytes, verifying that quercetin could reduce EGFR and ERK1/2 phosphorylation and inhibit EGFR signaling pathway activation, alleviating podocyte apoptosis. The administration of quercetin significantly reduced the pro-apoptotic protein Bax and Cleaved caspase-3, while the anti-apoptotic protein Bcl-2 was significantly upregulated (Liu Y. et al., 2022). Quercetin treatment has also been shown to reduce high early apoptosis levels to those observed in nondiabetic animals (Gomes et al., 2014). Another study employing *in vivo* and *in vitro* diabetes models verified that quercetin could prevent DKD by inhibiting the apoptosis of tubular epithelial cell through the PI3K–AKT pathway (Liu et al., 2024).

### 4.2 Activating and promoting autophagy

Autophagy is a highly conserved intracellular catabolic process that can regulate systemic blood glucose and induce podocyte differentiation in mammals (Parzych and Klionsky, 2014). Therefore, targeted autophagy is a potential novel therapeutic approach for DKD (Yasuda-Yamahara et al., 2015). Quercetin activates autophagy in diabetic mice, promotes podocyte differentiation through Notch pathway, and protects against podocyte damage in diabetic podocyte injury. Quercetin can also significantly reverse the significant decrease in LC3-II/LC3-I expression levels and the significant increase in p62 protein expression levels in diabetic mice (Zhu et al., 2024b). Guo et al. established the Goto Kakizaki rat model of diabetes, and found that quercetin treatment upregulated the expression of autophagy and pathway related proteins, such as LC3A/LC3B, Beclin-1, Pink-1, and Parkin. Conversely, it downregulated P62, PI3K, P-PI3K, AKT, P-AKT, mTOR, and P-mTOR. These results confirm that quercetin significantly improves kidney damage in Goto Kakizaki rats, possibly by inhibiting the PI3K/AKT/mTOR pathway and promoting autophagy (Guo et al., 2024).

**FIGURE 7:**
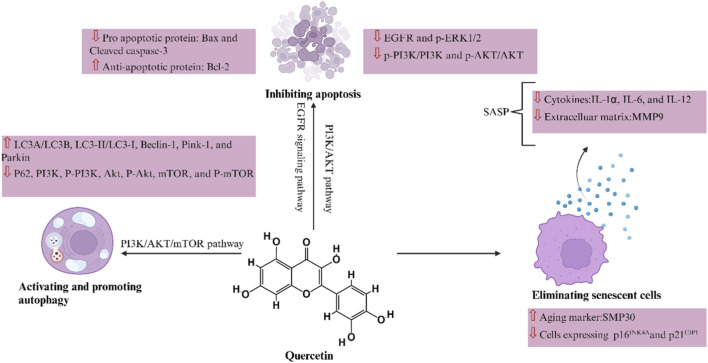
Mechanism of quercetin in the treatment of diabetic kidney disease: cell death.

Therefore, quercetin affects cell death through multiple pathways, including eliminating senescent cells, inhibiting apoptosis, and activating and promoting autophagy. The molecular mechanism of action of quercetin is shown in Figure 7; Table 2.

**TABLE 2:** Mechanism of quercetin in the treatment of diabetic nephropathy.

Quercetin dosage	Administration method	Model	Regulatory pathway	Effect	References
15 and 20 mg/kg	I.P injection for 6 weeks	45 mg/kg STZ induced diabetic rats	Cellular senescence	1. Average value of SMP30 protein significantly increased 2. Highest decrease in MG level.	Abharzanjani and Hemmati (2021)
150 mg/(kg•bw•d)	Oral gavage for four consecutive weeks	Maintain an HFD for 6 weeks and 50 mg/kg STZ I.P injection for 5 days to induce T2DM mice HK-2 cells were cultured under high-glucose.	PI3K/AKT pathway Apoptosis	1. Ameliorated blood glucose 2. Prevented the progression of DKD 3. mRNA expression of Bax and caspase3 markedly decreased 4. Ratio of Bax/Bcl-2 significantly decreased 5. Inhibited renal tubular cell apoptosis 6. Significantly reduced the ratios of p-PI3K/PI3K and p-AKT/AKT.	Liu et al. (2024)
10 mg/kg	Six weeks after STZ injections, oral quercetin per day for 4 weeks	I.P injection of STZ (100 mg/kg/day) for 3 days to induce T2DM mice	Apoptosis Oxidative stress.	1. Reduced polyuria and glycemia 2. Abolished hypertriglyceridemia 3. Decreased proteinuria and high plasma levels of uric acid, urea and creatinine 4. Reduced the high levels of early apoptosis 5. Significantly reduced levels of oxidative stress.	Gomes et al. (2014)
50 mg/kg, 100 mg/kg, and 150 mg/kg	Intragastric administration for 8 weeks	Genetically diabetic mice Mouse podocytes were cultured under high-glucose.	EGFR pathway Apoptosis	1. Protected renal function 2. Protected against glomerular injury 3. Significantly reduced Bax and cleaved Caspase-3, upregulated Bcl-2 4. Prevented glomerular podocyte apoptosis 5. Downregulated expressions of p-EGFR and p-ERK.	Liu et al. (2022b)
263.5 μg/mL	Received for 24 h	Rat kidney epithelial cells received STZ for 24 h	Apoptosis Anti-inflammation.	1. Significantly lowered the expression of TGF-β, TNF-α, and IL-6 2. Protective role in nephrotoxicity 3. Eliminated DPPH free radical 4. Protect cellular apoptosis 5. Anti-inflammatory activity.	DH et al. (2024)
50 mg/kg	Gavage for 20 weeks	Genetically diabetic mice Mouse podocytes were cultured under high-glucose.	Autophagy Notch pathway	1. Improved renal function 2. Improved glomerular injury, pathological changes, and renal fibrosis levels 3. Reduced podocyte dedifferentiation 4. Activated autophagy 5. Increased expression levels of LC3II/LC3I, decreased expression level of p62 6. Downregulation of the expression of NICD and p62, upregulation of the expression of LC3II/LC3I, synap, podocin and nephrin.	Zhu et al. (2024b)
50 and 75 mg/kg	Oral administration for 8 weeks	Rats were given a HFD for 10 weeks	PI3K/AKT/mTOR pathway Autophagy	1. Reduced blood sugar levels 2. Improved glycated serum protein levels and liver function 3. Improved lipid metabolism abnormalities and reduce blood lipid levels 4. Reduced the extensive expression of AKT, P62, and mTOR 5. Downregulated P62, PI3K, P-PI3K, AKT, P-AKT, mTOR, and P-mTOR 6. Upregulated expressions of LC3A/LC3B, Beclin-1, Pink-1, and Parkin.	Guo et al. (2024)
25, 50 and 100 mg/kg	Gavage once daily for the subsequent 7 weeks	I.P 55 mg/kg STZ in a dark environment to induce diabetes rats	NLRP3 inflammasome Anti-inflammation	1. Regulated renal urate transport-related proteins to reduce hyperuricemia 2. Regulated lipid metabolism-related genes to alleviate kidney lipid accumulation 3. Suppressed renal NLRP3 inflammasome activation 4. Downregulated rNLRP3, rASC, IL-1β, IL-18, and rCaspase-1 5. Protected kidney injury.	Wang et al. (2012)
50 mg/kg	Intragastrical administration per day for 8 weeks	Genetically diabetic mice HK-2 cells were cultured under high-glucose.	Anti-inflammation IL-6/STAT3 pathway.	1. Bound to YY1 and decreased its protein expression 2, reduced serum levels of IL-1β and TNF-α 3. Decreased protein expression of F4/80, p-STAT3 and IL-6.	Yang et al. (2023b)
10 and 20 μM	Added to the culture medium for 48 h	HMCs cells were cultured under high-glucose	miR-485-5p/YAP1 pathway Anti-inflammation Oxidative stress Cell proliferation.	1. Suppressed the levels of TNF-α, IL-1β, IL-6, and MDA 2. Promoted the secretion of SOD and GSH-px 3. Upregulated miR-485-5p and inhibited YAP1 expression.	Wan et al. (2022)
50 and 100 mg/kg	Oral administration for 1 week	I.P 60 mg/kg STZ induce diabetes rats	TGF-β1/Smad pathway Oxidative stress.	1. Reduced the kidney-to-body weight ratio 2. Improved kidney function and renal histopathological changes 3. Increased SOD and GSH levels and decreased MDA 4. Ameliorated oxidative stress 5. Increased nephrin, podocin, and Smad7 expression and decreased desmin expression 6. Decreased expression of TGF-β1, p-Smad2, and p-Smad3.	Gao et al. (2018)
25 mg/kg/day	I.P single dose for 30 days	I.P 45 mg/kg STZ induce diabetes rats	Oxidative stress	1. Reduced blood glucose levels 2. Decreased MDA levels and BUN 3. Increased SOD and CAT 4. Reduced renal histopathological changes.	Elbe et al. (2015)
10 mg/kg	Single abdominal subcutaneous injection daily for 8 weeks	Administrated HFD for 10 weeks and I.P 35 mg/kg STZ once	Oxidative stress Anti-inflammation.	1. Improved renal histopathological changes and inflammatory cells infiltration 2. Reduced BUN, MDA, TG, urine protein, albumin/creatinine ratio, and Scr level 3. Increased SOD 4. Suppressed neutrophil adhesion 5. Decreased ICAM-1 expression.	Tong et al. (2017)
50 mg/kg/day	Intragastrical administration per day for 8 weeks	I.P 60 mg/kg STZ induce diabetes rats	Oxidative stress	1. Decreased BUN, Scr, TNF-α, IL-1β, AGEs, and MDA level 2. Improved renal histopathological changes and biochemistry.	Tang et al. (2020a)

Abbreviation: LP, Intraperitoneal injection; MG, methylglyoxal; STZ, streptozotocin; P, phospho; T2DM, human proximal tubular epithelial cell (HK-2) type 2 diabetes mellitus; HFD, high-fat diet; DKD, diabetic kidney disease; DPPH, 2,2-diphenyl-1-picrylhydrazyfree assay; HMCs, human mesangial cells; MDA, malondialdehyde; SOD, superoxide dismutase; GSH, glutathione; BUN, blood urea nitrogen; CAT, catalase activities; Scr, serum creatinine; TG, triglyceride.

### 4.3 Reducing reactive oxygen species and oxidative stress

Oxidative stress (OS) plays a prominent role in DKD onset and progression. OS is defined as the excessive accumulation of ROS due to an imbalance between oxidants and antioxidants. Excessive ROS production beyond self-regulation processes leads to irreversible damage to cellular function or death (Rotariu et al., 2022). Chronic hyperglycemia induces OS, promotes the production of excessive ROS, and induces OS-related damage in the DNA and proteins involved in glomerular capillary and renal tubular structure and function, thereby exacerbating renal and systemic damage (Jin et al., 2023). Elbe et al. demonstrated that quercetin could prevent OS in streptozotocin (STZ)-induced diabetes rats by reducing lipid peroxidation and increasing the activities of superoxide dismutase (SOD) and catalase (CAT) (Elbe et al., 2015). In another study, the long-term oral administration of quercetin was found to reduce STZ-induced OS in DKD mice by reducing ROS levels (Gomes et al., 2014). In addition, quercetin could reduce the levels of OS markers (MDA and AGEs) and markers of inflammation (TNF-α and IL-1β) in the serum and kidney of STZ-induced DKD rats (Tang L. et al., 2020). Quercetin as a dietary supplement may play an antioxidant role in preventing or treating diabetes complications (Hu et al., 2021). Quercetin has been shown to restrict inflammatory cell infiltration, downregulate the expression of intercellular adhesion molecule-1 (ICAM-1), and reduce OS-related renal damage (Tong et al., 2017). Gao et al. confirmed quercetin administration significantly reduced OS and decreased the expression of podocyte injury marker desmin, thereby ameliorating podocyte injury in DKD rats (Gao et al., 2018).

### 4.4 Alleviating inflammation through multiple pathways

Inflammation is activated under harmful conditions, and chronic activation of the inflammatory response triggers collateral injurious effects (Goldszmid and Trinchieri, 2012). Regulating the inflammatory response has become a potential strategy for reducing DKD (Wada and Makino, 2016; Rayego-Mateos et al., 2020). Furthermore, inflammation is the most common source of chronic irritation leading to cellular senescence. Studies have shown that chronic low-grade inflammation increases with aging (Bektas et al., 2018). Ribeiro et al. found that indole phenol sulfate can induce low-grade inflammation through macrophages and promote the senescence of renal tubular epithelial cells during injury (Ribeiro et al., 2023). In addition, SASPs can create a complex proinflammatory environment that varies according to senescent cell type and may have numerous effects (Jun and Lau, 2010; Storer et al., 2013; Demaria et al., 2014; Fan et al., 2024).

**FIGURE 8:**
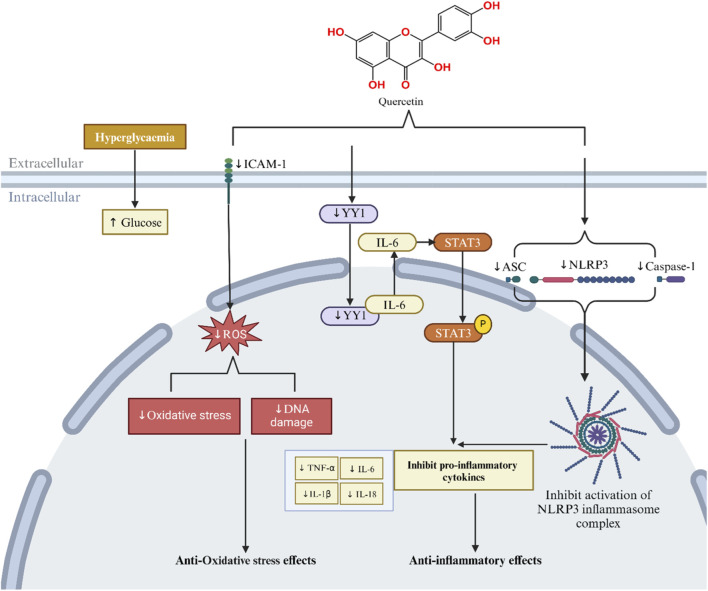
Mechanism of quercetin in the treatment of diabetic kidney disease: inflammation and oxidative stress.

Quercetin reduces the levels of proinflammatory cytokines (TNF-α and IL-1β) in the serum of db/db mice and HK-2 cells cultured in HG medium. Furthermore, it binds to Yin Yang 1 (YY1), reducing its protein expression, and regulates the IL-6/STAT-3 pathway. YY1 regulates the IL-6/STAT-3 pathway and is one of the specific mechanisms by which quercetin exerts antitubulointerstitial inflammation both *in vivo* and *in vitro* (Yang T. et al., 2023). Quercetin has been found to alleviate HG-induced inflammation in human mesangial cells by regulating the miR-485-5p/YAP1 axis. Specifically, it upregulates miR-485-5p expression, leading to YAP1 inhibition (Wan et al., 2022). In addition, quercetin has been shown to reduce the inflammatory response of kidney cells, and its administration reduced the expression of proinflammatory cytokines (TNF-α, and IL-6) (DH et al., 2024). Wang et al. further confirmed the anti-inflammatory effect of quercetin in STZ-induced DKD rats: quercetin significantly inhibited the overexpression of renal inflammasome components NLRP3, ASC, and Caspase-1, reduced the levels of proinflammatory cytokines (IL-1β and IL-18) in the serum and kidneys, and inhibited the activation of the NLRP3 inflammasome, thus protecting against kidney injury (Wang et al., 2012). The IL-1 signaling pathway mediated by the NLRP3 inflammasome can control the expression of SASPs and lead to senescence (Acosta et al., 2013). Overall, quercetin can inhibit proinflammatory factors and suppress inflammation in the treatment of DKD. However, no studies have been conducted on the association between quercetin and inflammation in the context of anti-senescence effects in DKD treatment. The mechanism by which quercetin exerts anti-inflammatory effects and inhibits cellular senescence in the treatment of DKD is most likely related to the NLRP3 inflammasome-mediated IL-1 signaling pathway. Overall, quercetin treatment mainly involves inhibiting proinflammatory cytokines and reducing ROS and OS through multiple pathways (Figure 8; Table 2).

## 5 Conclusions and future prospects

Quercetin has a clear therapeutic effect in DKD. In this review, we emphasized the role of quercetin in targeted cellular senescence therapy for DKD, aiming to identify hub targets and key pathways. Furthermore, we comprehensively reviewed the role of quercetin in relation to hub targets and key pathways.

Quercetin plus dasatinib effectively eliminates senescent cells in DKD. This review combined network pharmacology with a literature review. We ultimately screened 61 potential targets of quercetin in treating cellular senescence in DKD through multiple databases. Subsequently, we identified eight hub targets: AKT1, EGFR, ESR1, SRC, MMP9, GSK3β, MMP2, and PARP1. GO analysis revealed associated BPs and MFs, including oxidative stress response, cellular response to oxidative stress, response to reactive oxygen species, protein serine/threonine kinase activity, protein tyrosine kinase activity, and phosphatase binding. KEGG analysis demonstrated the involvement of the PI3K/AKT signaling pathway, MAPK signaling pathway, cell apoptosis, autophagy in animals, cell aging and other signaling pathways. On this basis, we further summarized the role of quercetin in key pathways and hub targets. In DKD animal and cell models, quercetin could eliminate senescent cells, inhibit apoptosis, activate and promote autophagy, reduce ROS and oxidative stress, and alleviate inflammation through various pathways. Quercetin could also inhibit the activation of the PI3K/AKT/GSK-3β pathway, reduce the activity of MMP9 and MMP2, and inhibit the EGFR signaling pathway. There is still a lack of direct experimental evidence regarding the specific roles of SRC, ESR1 and PARP1 in the cellular senescence process of DKD, as well as how quercetin precisely regulates these targets.

Research on the relationship between quercetin and cellular senescence is limited; thus, further *in vitro* and *in vivo* studies are needed to clarify the therapeutic effect of quercetin in DKD in terms of senescent cell clearance and the specific roles of quercetin in hub target and key pathway interactions. Gene knockdown or overexpression experiments in animal or cell models are needed to investigate the functional role of the identified hub genes in the quercetin treatment of cellular senescence in DKD. For example, as an upstream regulatory factor, the role of SRC in the balance between senescence and apoptosis needs to be clarified through gene knockout or overexpression experiments using DKD cells or animal models. Given that ESR1 may have crosstalk with pathways such as PI3K/AKT, the research should focus on whether quercetin affects downstream key signaling pathways such as PI3K/AKT/GSK-3β by regulating ESR1. For PARP1, research should focus on the regulatory mechanism of quercetin and evaluate the therapeutic potential of targeting PARP1 in improving renal function in DKD.

Due to limitations such as low quercetin bioavailability and solubility, along with disease complexity, few clinical studies have been conducted on the treatment of DKD with quercetin. Therefore, several clinical *in vivo* studies should be conducted to explore the long-term safety, clinical efficacy, and mechanism of quercetin in treating DKD. Recently, an increasing number of studies on DKD cellular senescence have shown that the autophagy pathway can effectively clear senescent cells. In the kidneys, autophagy deficiency in podocytes also appears to promote aging (Tang C. et al., 2020). The activation of autophagy can mediate the protective and antisenescence effects on podocytes (Li et al., 2023). Quercetin can activate autophagy and clear senescent cells; however, the relationship between these two effects is unknown. The future challenge is to study the crosstalk between aging and autophagy in a DKD model to investigate the potential mechanism of action of quercetin.
