# Supplementary material for: Multidimensional mechanisms of quercetin in diabetic kidney disease
Source: Front Cell Dev Biol. 2026 Feb 25;13:1705601. doi: 10.3389/fcell.2025.1705601 (PMC12975762; doi:10.3389/fcell.2025.1705601)
Supplement: Supplementary file 2 [file DataSheet1.docx]

Supplementary Material

# Materials and Methods of network pharmacology

## Collection and screening of targets for quercetin

To collect the targets of quercetin, we used PubChem database (https://pubchem.ncbi.nlm.nih.gov/) to obtain the SDF structure and SMILES of quercetin. Then we retrieved its targets through Swiss Target Prediction (http://www.swisstargetprediction.ch/) using SMILES, screening indicators: Probability*(P) ≥ 0.1 (Daina et al., 2019). And we predicted potential targets by inputting SDF structure into the PharmMapper database (http://www.lilab-ecust.cn/pharmmapper/) screening indicators: Norm Fit value of ≥0.8 (Liu et al., 2010). Finally, we used the Universal Protein database (Uniprot, http://uniprot.org/) to verify the targets (Apweiler et al., 2004).

## Collection of DN and cellular senescence related targets

Our study collected the DN related targets by searching the Genecards (https://www.genecards.org/) and Online Mendelian Inheritance in Man (OMIM, https://omim.org/#) through ‘diabetic nephropathy’ as key words (Amberger et al., 2015; Safran et al., 2022). Then, we merged the results and removed duplicate targets. The cellular senescence related targets were obtained using Genecards and Gene Set Enrichment Analysis (https ://www.gsea-msigdb.org/) database (Subramanian et al., 2005).

## ‘Quercetin-target-cellular senescence-disease’ network construction

The Excel software was used to screen the intersected relevant targets for quercetin, DN-related targets, and cellular senescence-related targets. Then, the ‘quercetin-target-cellular senescence-disease’ network was constructed through the Cytoscape 3.9.1. This network took diseases, drugs, components, and related targets as nodes, and their interrelationships as edges.

## Protein-protein interaction network

We used the String database (https://string-db.org) to construct the PPI network of the intersection targets through setting the species as Homo sapiens and the minimum required interaction score to 0.40 (Szklarczyk et al., 2021). The hub genes were screened by the five algorithms in CytoHubba plugin. And UpSet in R software was used to identify the intersection of the hub genes.

## GO and KEGG analysis

The Gene ontology (GO) and Kyoto encyclopedia of genes and genomes (KEGG) enrichment analysis were performed by gmt files downloaded from the GSEA platform (http://www.gsea-msigdb.org/). The Cytoscape 3.9.1 was used to construct the quercetin -target-pathway network. The GO enrichment results and KEGG pathway analysis results were visualized using the R software.

## Molecular docking of the quercetin and hub targets

We obtained the 3D structures of quercetin and hub genes from the Pubchem and Protein Data Bank (PDB) (http://www.rcsb.org/) database (Berman et al., 2000). Molecular docking was performed using Autodock Vina to validate the interaction of quercetin with feature genes, and the best four combinations of docking were selected and visualized using Pymol.

## R relevant code of GO and KEGG analysis

library("org.Hs.eg.db")

inputFile="Drug_Disease.txt"

outFile="id.txt"

rt=read.table(inputFile,sep="\t",check.names=F,header=F)

genes=as.vector(rt[,1])

entrezIDs <- mget(genes, org.Hs.egSYMBOL2EG, ifnotfound=NA)

entrezIDs <- as.character(entrezIDs)

out=cbind(rt,entrezID=entrezIDs)

colnames(out)=c("symbol","entrezID")

write.table(out,file=outFile,sep="\t",quote=F,row.names=F)

library("clusterProfiler")

library("org.Hs.eg.db")

library("enrichplot")

library("ggplot2")

library("pathview")

pvalueFilter=0.05

qvalueFilter=0.05

rt=read.table("id.txt",sep="\t",header=T,check.names=F)

rt=rt[is.na(rt[,"entrezID"])==F,]

colnames(rt)[1]="Gene"

gene=rt$entrezID

colorSel="pvalue"

if(pvalueFilter>0.05){

colorSel="qvalue"

}

kk <- enrichKEGG(gene = gene, organism = "hsa", pvalueCutoff =1, qvalueCutoff =1)

KEGG=as.data.frame(kk)

KEGG$geneID=as.character(sapply(KEGG$geneID,function(x)paste(rt$Gene[match(strsplit(x,"/")[[1]],as.character(rt$entrezID))],collapse="/")))

KEGG=KEGG[(KEGG$pvalue<pvalueFilter & KEGG$qvalue<qvalueFilter),]

write.table(KEGG,file="KEGG.txt",sep="\t",quote=F,row.names = F)

showNum=20

if(nrow(KEGG)<showNum){

showNum=nrow(KEGG)

}

pdf(file="barplot.pdf",width = 9,height = 11)

barplot(kk, drop = TRUE, showCategory = showNum, color = colorSel)

dev.off()

pdf(file="bubble.pdf",width = 9,height = 9)

dotplot(kk, showCategory = showNum, orderBy = "GeneRatio",color = colorSel)

dev.off()

**Reference:**

Amberger, J. S., Bocchini, C. A., Schiettecatte, F., Scott, A. F., & Hamosh, A. (2015). OMIM.org: Online Mendelian Inheritance in Man (OMIM®), an Online catalog of human genes and genetic disorders. *Nucleic Acids Research*, *43*(D1), D789–D798. https://doi.org/10.1093/nar/gku1205

Apweiler, R., Bairoch, A., Wu, C. H., Barker, W. C., Boeckmann, B., Ferro, S., Gasteiger, E., Huang, H., Lopez, R., Magrane, M., Martin, M. J., Natale, D. A., O’Donovan, C., Redaschi, N., & Yeh, L. S. L. (2004). UniProt: The universal protein knowledgebase. *Nucleic Acids Research*, *32*(DATABASE ISS.). https://doi.org/10.1093/nar/gky092

Berman, H. M., Westbrook, J., Feng, Z., Gilliland, G., Bhat, T. N., Weissig, H., Shindyalov, I. N., & Bourne, P. E. (2000). The Protein Data Bank. In *Nucleic Acids Research* (Vol. 28, Issue 1). http://www.rcsb.org/pdb/status.html

Daina, A., Michielin, O., & Zoete, V. (2019). SwissTargetPrediction: updated data and new features for efficient prediction of protein targets of small molecules. *Nucleic Acids Research*, *47*(W1), W357–W3664. https://doi.org/10.1093/nar/gkz382

Liu, X., Ouyang, S., Yu, B., Liu, Y., Huang, K., Gong, J., Zheng, S., Li, Z., Li, H., & Jiang, H. (2010). PharmMapper server: A web server for potential drug target identification using pharmacophore mapping approach. *Nucleic Acids Research*, *38*(SUPPL. 2). https://doi.org/10.1093/nar/gkq300

Safran, M., Rosen, N., Twik, M., BarShir, R., Stein, T. I., Dahary, D., Fishilevich, S., & Lancet, D. (2022). The GeneCards Suite. In *Practical Guide to Life Science Databases* (pp. 27–56). Springer Nature. https://doi.org/10.1007/978-981-16-5812-9_2

Subramanian, A., Tamayo, P., Mootha, V. K., Mukherjee, S., Ebert, B. L., Gillette, M. A., Paulovich, A., Pomeroy, S. L., Golub, T. R., Lander, E. S., & Mesirov, J. P. (2005). *Gene set enrichment analysis: A knowledge-based approach for interpreting genome-wide expression profiles*. www.pnas.orgcgidoi10.1073pnas.0506580102

Szklarczyk, D., Gable, A. L., Nastou, K. C., Lyon, D., Kirsch, R., Pyysalo, S., Doncheva, N. T., Legeay, M., Fang, T., Bork, P., Jensen, L. J., & von Mering, C. (2021). The STRING database in 2021: Customizable protein-protein networks, and functional characterization of user-uploaded gene/measurement sets. *Nucleic Acids Research*, *49*(D1), D605–D612. https://doi.org/10.1093/nar/gkaa1074
